# Hospitals in Australia

**Published:** 1894-11-03

**Authors:** 


### HOSPITALS IN AUSTRALIA.

The new buildings for the Sydney Hospital, which have been standing unfinished for more than a decade, are at last an accomplished fact, and they were opened on Friday, August 10th, by Sir George Dibbs, the ex-Premier, who was finally instrumental in having the place completed, despite the fact that on more than one occasion, he was some years past, when in former Governments, a factor in the arrest of its progress, owing to a disagreement as to the necessity of a large general hospital in its position. The bulk of evidence was against such a large building being erected there, but now it is a magnificent structure, and will doubtless be productive of good work.

The hospital is in three sections, two wings, and a main structure. The main building is designed on the pavilion principle. In the wards an average air space of 1,700 cubic feet is arranged for each bed. The lighting might be better, but the ventilation is good. The sanitary arrangements are good. The new building contains eight wards, accommodating

180 beds, and the old southern wing contains 70 beds, making 250 beds altogether. In the central block there is accommodation for resident medical officers. On the ground floor are visiting medical officers' rooms, dispensary and examination room for patients. On the first floor is lobby and large waiting-room, leading from Macquarie Street. The main front is of freestone, all other walls brick and cemented. Fire proof material is used in all floors, and in addition there is a fire hose on every floor. Water towers are provided with cisterns containing 10,000 gallons, as a stand-by in case of fire. The whole area of the site is three acres.

The foundation stone of the new building was laid in 1881 by the then Governor, Lord Augustus Loftus. The Sydney Hospital was established 85 years ago under Governor Macquarie, and until some two years ago a miserable, unsafe, wooden tenement constituted the main part of the hospital. At this time it was deemed unsafe to occupy these buildings for any further period, and the nurses' quarters, known as the Nightingale Wing, was temporarily fitted up, which, with the southern wing before alluded to, has done duty as a hospital pending the completion of the present buildings, the nurses being accommodated in premises close by.

To show the number of patients attended to at this hospital, it might be noted that 3,083 were treated indoor, and there were 106,533 separate attendances in the outdoor department during the year 1893.

The Western Suburbs Cottage Hospital was opened on August 16th by His Excellency Sir Robert Duff, the Governor of New South Wales. It is meant to act as a place for the reception of accidents and emergency cases for these suburbs, being centrally situated and distant about five miles from the nearest hospital, viz., the Prince Alfred. The movement to establish it was commenced in 1891, and the foundation stone was laid in 1893. The institution provides for twelve beds and a child's cot. The expenditure, with equipment, would reach about £4,200, towards which £4,000 has been contributed. The hospital was built from plans prepared by Mr. J. E. Ellis, of 295, Pitt Street, Sydney, whose design was chosen from a number of competitors. The type of architecture is known as English Domestic. The area of the site, situate at corner of Liverpool-road and Brighton-street, Enfield, is two acres. The land has a gentle slope from north to east. The ward block is situate in the centre of groups of buildings, the operating and board-rooms in front and administrative block at the rear. The ward block is divided into two portions, one for male and the other for female patients. The uniform height of the rooms is 11 feet. Two wards measure 27 by 24 feet, each containing 6 beds; operating-room, 21 by 14 feet; committee-room, 14 by 20 feet, with bow window 9 by 3 feet Running round the ward and front blocks and connecting the various portions of the establishment are verandahs 7 feet in width. The material used in construction is brick on rubble foundations; roofs are of slate, and the ceilings of the wards are corrugated iron, painted. Mr. J. E. Ellis as architect, and Messrs. McKellan and Wilson as contractors, were congratulated by the Building Committee upon the faithful, workmanlike, and expeditious manner in which the work had been carried out.

